# Reverse Total Shoulder Arthroplasty in Patients with Os Acromiale: A Systematic Review of Clinical and Radiographic Outcomes

**DOI:** 10.3390/jcm14113935

**Published:** 2025-06-03

**Authors:** Riccardo Ranieri, Matthias Schroeder, Juan David Lacouture, Ciro Tatangelo, Giacomo Delle Rose, Marco Conti, Raffaele Garofalo, Alessandro Castagna

**Affiliations:** 1 Department of Biomedical Sciences, Humanitas University, Via Rita Levi Montalcini 4, 20090 Milan, Italy; 2 IRCCS Humanitas Research Hospital, Via Manzoni 56, 20089 Milan, Italy; 3 Serena Del Mar Hospital, m.8 Via al Mar Serena del Mar, Cartagena de Indias 130002, Bolivar, Colombia; 4 Shoulder and Sport Medicine Unit, Miulli Hospital, Strada Prov. 127 Acquaviva—Santeramo Km, 4, 70021 Acquaviva delle Fonti, Italy

**Keywords:** os acromiale, reverse shoulder arthroplasty, acromion

## Abstract

**Background/Objectives**: This study aims to conduct a systematic review to determine the clinical and radiographic outcomes and postoperative complications of reverse total shoulder arthroplasty (RTSA) in patients with os acromiale. Methods: A systematic review was conducted according to PRISMA guidelines. Studies investigating outcomes of RTSA in patients with os acromiale were included. Data regarding prevalence, clinical outcomes, range of motion, complications, and radiographic findings was extracted. **Results**: Six studies were included involving a total of 161 patients with os acromiale who received an RTSA. Os acromiale was present in approximately 6.4% of the total RTSA cases. Comparative studies reported no significant differences in clinical outcomes, complication rates, or reoperations between patients with and without os acromiale. One study with consecutive follow-up evaluations reported postoperative acromial tenderness in up to 27% of patients, which resolved spontaneously in most cases. Radiographic inferior displacement of the os acromiale occurred in 54% (range: 28–63%) of cases, but did not correlate with worse functional outcomes. **Conclusions**: Os acromiale does not represent a contraindication to RTSA and does not significantly compromise clinical outcomes. Radiographic acromial tilt often occurs without clinical relevance. Preventive surgical fixation of the os acromiale is not routinely recommended. Nonetheless, acromial tenderness may be present postoperatively, with a high likelihood of spontaneous symptom resolution.

## 1. Introduction

Os acromiale represents a failure of fusion of the acromial apophysis [1,2]. There are three ossification centers: meta-acromion, meso-acromion, and pre-acromion [1,3,4]. Normally, these centers fuse by early adulthood, with the basi-acromion integrating with the scapular spine around age 12, and the remaining centers completing fusion between ages 15 and 18 [1,2,4,5]. The average incidence is ~8% with a range of 1–15% being reported, varying among different studies, and it is often detected incidentally on radiographs, CT scans, or MRIs [3,4].

While frequently asymptomatic, it has been associated with pathologic conditions, including subacromial impingement, rotator cuff pathology, and chronic shoulder pain due to deltoid-induced mechanical stress on the unfused acromial segment [2,3,6].

Reverse total shoulder arthroplasty (RTSA) restores the shoulder function by medializing the center of rotation and distalizing the humerus. This leads to an increase in deltoid tension, and as a result, the altered biomechanics may subject an unfused acromion to increased mechanical strain. This increased strain in the setting of RTSA may raise concerns about fragment migration, instability, pain, and reduced functional outcomes [7,8,9]. While some studies have reported no significant effect of os acromiale on postoperative function, others suggest potential biomechanical consequences, particularly related to fragment displacement or acromial stress fractures [10,11,12].

Despite the increasing number of RTSAs performed, the specific impact of os acromiale in these patients remains unclear. This systematic review aims to summarize current evidence on the influence of the os acromiale on clinical and radiological outcomes after RTSA, with a focus on potential complications, functional performance, and biomechanical considerations.

## 2. Materials and Methods

This systematic review was conducted in accordance with the PRISMA 2020 guidelines. A completed PRISMA checklist and a flow diagram are provided in the Supplementary Materials.

### 2.1. Eligibility Criteria

Clinical studies reporting outcomes of reverse total shoulder arthroplasty (RTSA) in patients with os acromiale were considered eligible, regardless of the level of evidence. Studies were included if they reported at least one of the following outcomes: functional results, radiological findings, or complications. Exclusion criteria were case reports, narrative reviews, non-English publications, and studies with insufficient clinical or imaging outcome data. Studies including patients with acquired acromial insufficiency (e.g., fractures and fragmentation) were considered only if data for os acromiale were reported separately.

### 2.2. Information Sources and Search Strategy

A systematic search was performed in PubMed, Embase, Google Scholar, and the Cochrane Library on 10 January 2025, using the following search terms: (“os acromiale”) AND (“reverse shoulder arthroplasty”).

No date restrictions were applied. Only English-language articles were considered. The reference lists of all included studies were manually screened to identify additional relevant publications.

### 2.3. Study Selection

**Figure 1:**
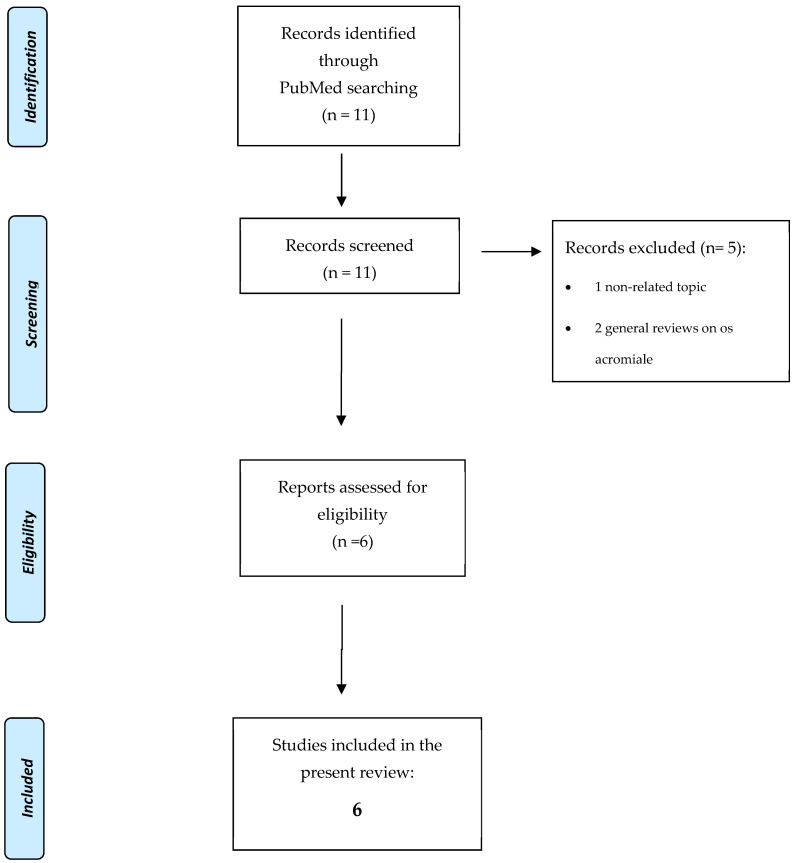
Flowchart illustrating study selection process according to PRISMA guidelines.

Two independent reviewers (M.S. and R.R.) screened the titles and abstracts using predefined eligibility criteria. Full texts were retrieved for all potentially eligible articles. Disagreements were resolved through discussion or consultation with a senior reviewer (A.C.). The PRISMA flow diagram (Figure 1) summarizes the study selection process.

### 2.4. Data Collection Process

Data were independently extracted by both reviewers and entered into a shared spreadsheet. The following variables were collected: study design, the number of patients with os acromiale, the classification of os acromiale, surgical details, follow-up durations, clinical outcomes, radiological findings, and complications. When available, results for patients with and without os acromiale were analyzed separately.

### 2.5. Risk of Bias Assessment

The methodological quality and outcome reporting of the included studies were assessed according to the recommendations proposed by Coleman et al. [13], which provide a structured framework for evaluating the design and reliability of clinical studies in orthopedic surgery.

## 3. Results

A total of six studies reporting outcomes of RTSA in patients with os acromiale were finally included in the present systematic review. The characteristics of the included studies are listed in Table 1. Among them, five were comparative studies, comparing outcomes between RTSA patients with or without os acromiale [10,11,12,14,15]. Three papers also included cases of acquired acromial insufficiency, consisting of preoperative acromial fracture or fragmentation, mainly due to advanced cuff tear arthropathy (CTA) [10,12,15]. If possible, only the data regarding patients with os acromiale were retrieved and presented for the specific purpose of the present systematic review. Considering the total series of RTSA patients, from which the patients with os acromiale were extracted, the prevalence of os acromiale in the setting of RSA is 6.4% (Table 1). The clinical and radiological outcomes are summarized in Table 2.

### 3.1. Qualitative Synthesis of Clinical and Radiological Results

Walch et al. [10], among 457 RTSA cases, identified 41 patients with mixed acromial pathology, including 23 with os acromiale. This study found that os acromiale did not negatively impact postoperative function, range of motion, or satisfaction. However, inferior acromial tilt occurred postoperatively at a rate of 63% considering all series, but it did not consistently correlate with functional decline. One case of os acromiale underwent operative fixation using a tension band, with subsequent radiological non-union, but a good clinical outcome. The authors concluded that os acromiale is not a contraindication for RTSA, and they discouraged the osteosynthesis of the fragment in the case of RTSA.

Aibinder et al. [16] conducted a retrospective analysis of 25 RTSA cases in patients with CTA and os acromiale, evaluating functional outcomes and postoperative complications. Their results demonstrated significant improvements in pain and functional outcomes. Inferior postoperative tilt occurred in 28% of cases at an average of 32.3°, but it did not appear to affect clinical outcomes or range of motion. Notably, only one patient required surgical excision of the os acromiale and deltoid muscle advancement due to persistent pain.

Ersen et al. [14], in a case–control study, investigated the incidence and impact of os acromiale in RTSA patients with CTA. Among 46 patients analyzed, 10 had meso-acromion-type os acromiale. While both groups showed significant improvements in Constant, Q-DASH, and VAS scores postoperatively, there were no significant functional differences between patients with and without os acromiale. Radiographic analysis revealed that the meso-acromion fragment tended to migrate distally postoperatively due to deltoid tension, leading to a shorter acromiohumeral distance. Despite this finding, no correlation was observed between fragment migration and functional outcomes.

Werner et al. [15] conducted a matched case–control study analyzing 44 RTSA cases, including 11 patients with preoperative acromial compromise (defined as os acromiale, acromial thinning, or fragmentation) who were matched to 33 controls. Two years postoperatively, there were no significant differences in ASES, SF-12, or Marx activity scale scores between groups. Furthermore, no patient experienced complications or required revision surgery. The authors concluded that preoperative acromial pathology, including os acromiale, does not compromise RTSA outcomes, supporting its safety and efficacy in these patients.

Carpeggiani et al. [11] conducted a cohort study analyzing 45 RTSA cases with os acromiale, which were selected from a total of 52 identified cases, and compared them to 133 controls without os acromiale. The study included patients with mixed indications for RTSA evaluated at different time points, with a mean last follow-up of 52 months (range 12–121 months). The os acromiale was classified as meso-acromion in 30 (67%) cases. The presence of os acromiale was associated with significantly reduced active flexion (104° vs. 114°) at a 1-year follow-up and abduction (103° vs. 121°) at 2 years postoperatively, though these differences were not observed at the last follow-up of 52 months, and overall Constant and Subjective Shoulder Value (SSV) scores were not significantly different between groups (CS 70 vs. 76; SSV 70% vs. 73%). The rate of postoperative complications and revision was not different between the two groups, and no complications directly caused by os acromiale were found. While 12 (27%) patients developed postoperative local tenderness at the os acromiale site, this symptom resolved in 8 out of the 12 (67%) patients without any treatment. This group of patients showed a significantly lower abduction at 2 years compared to patients with os acromiale and no tenderness, as well as a negative trend for the other functional scores. The authors concluded that, while RTSA reliably improves function in patients with os acromiale, there is a mild negative impact on the postoperative range of motion, and postoperative local tenderness at the os acromiale can be expected in one out of four patients, with spontaneous resolution in the majority of patients.

Davis et al. [12] conducted a retrospective analysis of 525 RTSA cases, including 72 shoulders with preoperative scapular stress fractures, of which 40 had os acromiale. Their study examined postoperative satisfaction and functional outcomes, comparing patients with and without preoperative fractures. While all groups showed improvement postoperatively, those with multifragment scapular fractures had significantly lower satisfaction rates, ASES scores, and higher pain at final follow-up. In contrast, patients with os acromiale achieved functional gains comparable to non-fractured cases, though with slightly lower satisfaction scores over time. These findings suggest that os acromiale does not compromise RTSA outcomes, but the condition described as multifragment fractures may require more cautious management.

### 3.2. Methodological Quality Assessment

We analyzed the included studies for methodological quality and outcome reporting using the criteria recommended by Coleman et al. [13]. Our assessment revealed a lack of uniformity in study design and outcome reporting standards across the available literature. The mean Coleman score for the studies included in this review was 57, with individual scores ranging from 47 to 70 points, indicating moderate methodological quality overall.

## 4. Discussion

The primary findings of this review suggest that clinical outcomes are not adversely affected in the setting of os acromiale, and therefore, the presence of os acromiale may not represent a contraindication for reverse total shoulder arthroplasty (RTSA).

Patients with os acromiale undergoing RTSA achieved good functional outcomes and patient-reported outcome measures (PROMs) comparable to those of patients without os acromiale [17,18,19]. None of the five comparative studies that were included reported significant differences in outcome measures at the final follow-up.

Only Carpeggiani et al. found significantly lower active elevation and abduction at 1- and 2-year follow-ups; however, these differences were no longer present at the final follow-up [11]. 

A similar conclusion can be drawn regarding the risk of postoperative complications and reoperations. The presence of os acromiale does not appear to have a negative impact with regard to postoperative complications or reoperation rates. Among the included studies, two reported a total of seven postoperative complications resulting in five revision surgeries, none of which appeared to be related to the presence of an os acromiale (two dislocations, two cases of glenoid loosening, and two scapular spine fractures) [11,16,20]. However, two studies highlighted the potential issue of postoperative tenderness and pain localized at the acromion [11,16]. In the study by Abinder et al. [16], one case of persistent acromial pain was treated with reoperation involving os acromiale excision and deltoid advancement, resulting in symptom resolution within six months. Similarly, Carpeggiani et al. reported 12 cases (27%) of postoperative acromial tenderness, which resolved spontaneously in 8 out of the 12 patients at the last follow-up [11]. Based on the studies included in the present review, this condition is reported infrequently, with only Carpeggiani et al. documenting a relevant incidence (27% at one year). This observation may be attributed to the fact that only this study conducted detailed evaluations of patients at multiple consecutive follow-up points, whereas the other studies assessed outcomes only at the final follow-up.

Interestingly, the study by Davis et al. [12], which also included progressive clinical assessments, found a significant difference in the ASES pain score at one year between patients with and without os acromiale. This may potentially be explained by the occurrence of postoperative acromial tenderness, which tends to resolve over time. In summary, persistent tenderness and pain localized at the acromion may occur at a non-negligible rate following RTSA in patients with os acromiale. However, symptoms appear to resolve spontaneously in most cases without the need for further reoperations, with good clinical outcomes observed at two years. Further studies are warranted to better define the incidence and natural course of this postoperative problem.

**Figure 2:**
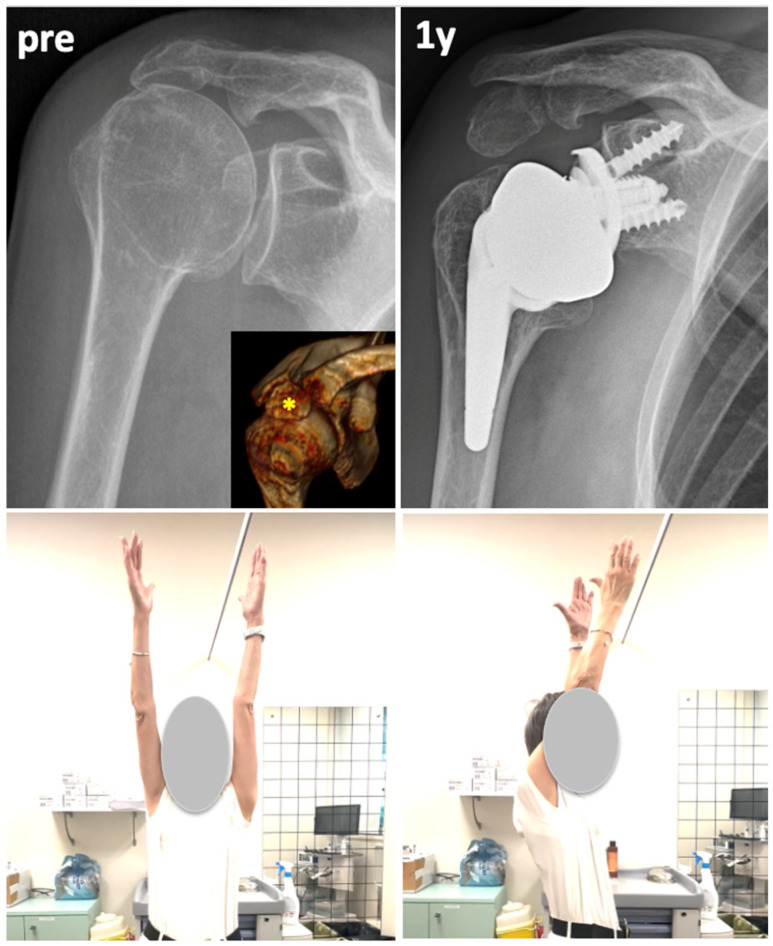
A case of RTSA in case of os acromiale (yellow * on the 3D CT scan). After 1 year from RTSA, the x-ray shows an evident inferior tilt of the meso-acromion with decreased acromiohumeral distance. Clinically, the patient recover good abduction and anterior flexion.

Another important finding of the present review is the relatively high rate of radiographic displacement of the os acromiale observed on postoperative X-rays, with an average incidence of 54% (range: 20–63%). However, none of the included studies reported a significant impact of this radiological finding on clinical outcomes [10,11,14,16]. This suggests that progressive inferior displacement of the os acromiale may occur following RTSA, likely due to increased deltoid tension and lengthening of the muscle fibers resulting from the distalization of the center of rotation [7,8,9,21]. Theoretically, a reduced acromion–humeral distance due to os acromiale displacement following RTSA could lead to early impingement of the greater tuberosity beneath the acromion, potentially limiting abduction and flexion [22,23,24]. However, all comparative clinical studies included in this review found that this phenomenon does not have a negative impact on functional outcomes or range of motion. A potential explanation for this finding is that, unlike the impingement between the greater tuberosity and a normal, fixed acromion, which can limit arm elevation [25] in the case of an os acromiale, contact between the greater tuberosity and the acromion may induce dynamic displacement of the os acromiale. This movement could help to increase the subacromial space during arm elevation, thereby compensating for the apparently reduced acromion–humeral distance seen radiographically (Figure 2). This theory, however, requires further validation through dynamic or fluoroscopic analyses of RTSA in patients with os acromiale.

Finally, an important question arises regarding the preventive fixation of the os acromiale during the RTSA procedure. Only Walch et al., in their series, reported a case of operative tension band fixation of the os acromiale, which resulted in non-union with persistent acromial tilt, although the patient achieved satisfactory clinical outcomes [10]. In agreement with this author, the findings of the present review discourage any additional procedures for os acromiale fixation when combined with RTSA.

This study has some limitations. Firstly, the quality of the included studies is low, with only levels of evidence III or IV and retrospective comparative designs. Secondly, some of these studies also included cases of acquired acromial insufficiency, defined as preoperative acromial fractures or fragmentation, primarily due to advanced CTA [10,12,15]. However, whenever possible, data from patients with os acromiale were analyzed and presented separately. Finally, the varying outcome measures used across studies (often at different follow-up time points) prevented the performance of a meta-analysis.

## 5. Conclusions

Os acromiale does not represent a contraindication to RTSA and is not associated with a higher risk of complications or reoperation. Patients with os acromiale undergoing RTSA achieved good functional outcomes and PROMs comparable to those of patients without os acromiale. Radiographic inferior displacement of the os acromiale may occur in over 50% of cases, but without a significant impact on clinical outcomes. Acromial tenderness may occur postoperatively, with a high likelihood of spontaneous symptom resolution after 2 years.

## Figures and Tables

**Table 1:** Synopsis of all the articles regarding RTSA in patients with os acromiale included in the present systematic review.

Publication	Comparative (LOE)	Preoperative Diagnosis	N^ Cases of RTSA + OS Acromiale (Total Cases If Mix Series)	Total RTSA Series with Same Indication (%)	Acromial Insufficiency Included	Os Acromiale Type (Pre/Meso/Meta)	Follow-Up (Months)
Walch et al. JSES 2009 [10]	Yes (III)	CTA, MCT, HA revision	23 (41)	457 (5%)	Yes	23 meso	**40 (24–100)**
Aibinder et al. JSES 2017 [16]	No (IV)	CTA, MCT	25	N/A; 1079 total RSA database	No	3:20:2	31 (24–81)
Ersen et al. OT:SR 2019 [14]	Yes (III)	CTA	10	46 (22%)	No	10 meso	60 (24–105)
Werner et al. HSSJ 2019 [15]	Yes (III)	CTA	N/A (11)	124 (9%)	Yes *	N/A	28
Carpeggiani et al. OJSM 2020 [11]	Yes (III)	Mix	45 reviewed; 52 os acromiale	962 (5%)	No	14:30:1	52 (12–121)
Davis et al. JSES 2024 [12]	Yes (III)	CTA, MCT, RA	40 (72)	525 (8%)	Yes	N/A	76 ± 80
**TOTAL or weighted MEAN**	-	-	161 136 ^	3193 2114 ^^ **(6.4%)**	-	-	**52 months**

^ For the prevalence count, all 11 patients from the study by Werner et al. [15] were considered, while the 25 patients in Abinder et al.’s [16] study were not considered. ^^ The number for the total RSA series with the same diagnosis was not available for Abinder et al. [16] so this study was not included in the prevalence count. Numeric variables are reported as mean ± st. dev. or mean (range) according to the publications. CTA = cuff tear arthropathy; MCTs = massive rotator cuff tears; HA = hemiarthroplasty; N/A = not available. * Data for Os Acromiale Not Reported Separately.

**Table 2:** Complications, reoperation, and clinical and radiological results for a patient with os acromiale undergoing an RTSA.

Publication	Postoperative Complications	Postoperative Problems	Revision/Reoperation	Clinical Results	Vs RTSA	Radiological Acromial Tilt, n° Cases (5)	Acromial Tilt Influence on Outcome	Radiological Notes
Walch et al. JSES 2009 [10]	N/A	N/A	No	CS 68 AF 141°	No differences	24/38 (63%) *	No	-
Aibinder et al. JSES 2017 [16]	2 dislocations	1 painful OSa 3 unsatisfactory results (no further explanation)	2 revisions 1 reoperation (OSa excision + deltoid advancement)	Pain 2.0 ASES 66 AF 124° ER1 46° IR L4	N/A	7/25 (28%)	No	Postoperative inferior acromial tilt = 32.3° (10–64)
Ersen et al. OT:SR 2019 [14]	No	No	No	Pain 1.2 CS 66.4 qDASH 22 AF 130° ER1 29°	No differences	N/A	No	AH distance = 19.3 (16–22) mm (significantly shorter than controls, 32.3 mm)
Werner et al. HSSJ 2019 [15]	No	No	No	ASES 69 SF-12 PCS 36.8 SF-12 MCS 54 Marx activity scale 6.5	No differences	N/A	N/A	-
Carpeggiani et al. OJSM 2020 [11]	2 glenoid loosening 2 spine fractures 1 not specified	12 painful OSa	3 revisions	Relative CS 70 SSV 70 AF 107° ER1 23°	Lower AF at 1 y (104° vs. 114°) and lower abduction at 2 y (103° vs. 121°) Slight negative trend after 2 y	27/45 (60%)	No	-
Davis et al. JSES 2024 [12]	N/A	N/A	N/A	Pain 1.7 ASES 64	No differences (excluding MF fracture) Lower ASES pain (33 vs. 39.2) at 1 y	N/A	N/A	-
**TOTAL or weighted MEAN**	7/161 (4.3%)	-	6 (3.7%)	-	-	58/108 (54%)	-	-

OSa = os acromiale, MFs = multifragments, ASES = American Shoulder and Elbow Surgeons Score; VAS = Visual Analog Scale; CS = Constant score; DASH = Disability of the Arm, Shoulder, and Hand; AF = anterior flexion; ER1 = external rotation in position 1; IR = internal rotation; SF-12 = 12-Item Short-Form Health Survey; PCS = physical component score; MCS = mental component score; AH = acromiohumeral. * Percentage was approximated for the total series.

## Supplementary Materials

The following supporting information can be downloaded at: https://www.mdpi.com/article/10.3390/jcm14113935/s1.
