# Supplementary material for: Reverse Total Shoulder Arthroplasty in Patients with Os Acromiale: A Systematic Review of Clinical and Radiographic Outcomes
Source: J Clin Med. 2025 Jun 3;14(11):3935. doi: 10.3390/jcm14113935 (PMC12156067; doi:10.3390/jcm14113935)
Supplement: Supplementary file 1 [file jcm-14-03935-s001.zip › PRISMA FLOW CHART.pdf]

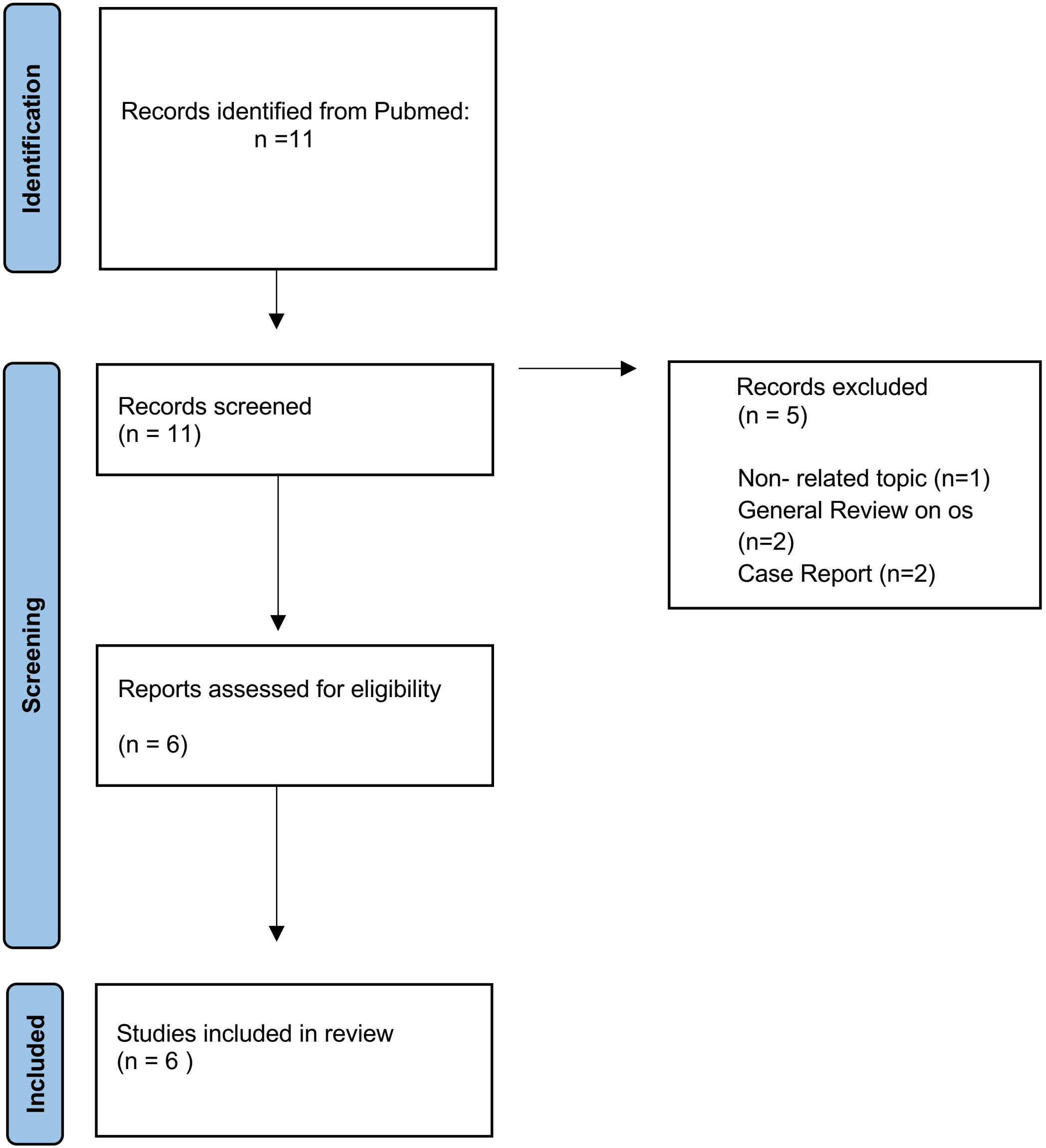
Identification
Records identified from Pubmed: n =11
Screening
Records screened (n = 11)
Records excluded (n = 5)
Non- related topic (n=1)
General Review on os (n=2)
Case Report (n=2)
Reports assessed for eligibility (n = 6)
Included
Studies included in review (n = 6)
